# Serum level of vitamin D3 in cutaneous melanoma

**DOI:** 10.1590/S1679-45082014AO3090

**Published:** 2014

**Authors:** Renato Santos de Oliveira, Daniel Arcuschin de Oliveira, Vitor Augusto Melão Martinho, Célia Beatriz Gianotti Antoneli, Ludmilla Altino de Lima Marcussi, Carlos Eduardo dos Santos Ferreira

**Affiliations:** 1 Hospital Israelita Albert Einstein, São Paulo, SP, Brazil.; 2 Hospital A.C. Camargo, São Paulo, SP, Brazil.; 3 Universidade Cidade de São Paulo, São Paulo, SP, Brazil.

**Keywords:** Calcifediol, Cholecalciferol, Receptors, calcitriol, Vitamin D deficiency, Melanoma, Immune system

## Abstract

**Objective:**

To compare the level of vitamin D3 in cutaneous melanoma patients, with or without disease activity, with reference values and with patients from a general hospital.

**Methods:**

The serum levels of vitamin D3 were measured in cutaneous melanoma patients, aged 20 to 88 years, both genders, from January 2010 to December 2013. The samples from the general group were processed at *Hospital Israelita Albert Einstein* (control group). Data analysis was performed using the Statistics software.

**Results:**

A total of 100 patients were studied, 54 of them men, with mean age of 54.67 years, and 95 Caucasian. Out of these 100 patients, 17 had active disease. The average levels of vitamin D3 in the melanoma patients were lower than the level considered sufficient, but above the average of the control group. Both groups (with or without active disease) of patients showed a similar distribution of vitamin D3 deficiency.

**Conclusion:**

Vitamin D3 levels in melanoma patients were higher than those of general patients and lower than the reference level. If the reference values are appropriate, a large part of the population had insufficient levels of vitamin D, including those with melanoma, or else, this standard needs to be reevaluated. No difference in vitamin D3 levels was found among melanoma patients with or without active disease. More comprehensive research is needed to assess the relation between vitamin D and melanoma.

## INTRODUCTION

Cutaneous melanoma is a malignant neoplasm that originates in melanocytes or their precursor cells, and which has a high potential for metastasizing. Although not the most frequent type of skin cancer, it is the most lethal. There is epidemiological evidence that solar radiation is related to its carcinogenesis. For the year 2014, a total of 5,890 new cases are expected in Brazil.^(1)^

The vitamin D receptor (VDR) is present in normal melanocytes and in certain cell lines of melanoma.^(2)^ Cholecalciferol or 25-hydroxyvitamin D is normally formed in the skin from 7-dehydrocholesterol in a photochemical reaction catalyzed by the ultraviolet component of solar light. Vitamin D3 is not biologically active, but is converted by hepatic and renal enzymes into 1,25-dihydroxycholecalciferol (vitamin D or calcitriol), a hormone that regulates the absorption of calcium in the bowel and its levels in the kidneys and bones.

Among other functions of vitamin D, there is one that is extremely important, that of strengthening the immune system. The effect of vitamin D on the immune system is translated as increased innate immunity, associated with a multifaceted regulation of acquired immunity. An association between deficient levels of vitamin D3 and various types of malignant neoplasms such as colon, breast, and skin cancer, has already been shown.^(3)^

There is ongoing controversy in the scientific community as to how much solar light is appropriate for the balance between the negative and positive effects of solar ultraviolet (UV) radiation exposure.

Solar exposure is essential for vitamin D to be produced by the skin, although exposure to UV radiation, without a doubt, is the most important environmental factor for all types of cancer in this location, since it causes DNA damage to skin cells. Many studies evaluated the important relation between solar light, vitamin D, and skin cancer.^(4,5)^

Melanoma is highly aggressive, immunogenic, and has the ability to modulate the immune system to its own advantage. The patient with melanoma presents with numerous cellular immune defects.^(6)^ Data suggest that the normal levels of vitamin D3 at the time of diagnosis are related to a better prognosis in patients with cutaneous melanoma.^(7,8)^

We present a cross-sectional study that compares the levels of vitamin D3 in patients with cutaneous melanoma, in activity or not, with normal levels recommended by clinical laboratories and with values obtained in patients of a general hospital.

## OBJECTIVE

To compare the serum level of 25-hydroxyvitamin D (vitamin D3) in patients with cutaneous melanoma with active and non-active disease with the values considered normal for the general population.

## METHODS

This study was duly analyzed and approved by the National Committee of Ethics in Research (CONEP), under CAAE # 18762713.9.0000.5492. All patients were instructed as to the study and signed the Informed Consent Form.

The serum levels of 25-OH-vitamin D were dosed in patients with cutaneous melanoma, aged 22 and 80 years, of both genders, from January 2010 to December 2013, treated and registered at a clinic specialized in skin cancer. As of January 2010, at this clinic, serum levels of 25-OH-vitamin D are routinely dosed upon admission of all patients with a diagnosis of melanoma, as well as during follow-up.

Data were collected on the levels of vitamin D3 from the medical charts identified with diagnoses of melanoma, as well as the state of activity or not of the disease in these patients. Vitamin D3 dosing was performed by automated immunoassay in serum samples. Reference values for vitamin D are determined by literature. Currently, serum levels >30ng/mL (nanograms per mL) are considered adequate. Values between 10 and 30ng/mL are insufficient and values <10ng/mL are considered as vitamin D deficiency.^(9)^ The control group was made up of general patients whose samples for vitamin D3 dosing were processed by the clinical laboratory of *Hospital Israelita Albert Einstein* (HIAE).

The activity of melanoma (at the primary site or in metastases) was evaluated by the clinical examination and by supplementary tests (computed tomography of the chest, ultrasonography of the upper abdomen, and lactic dehydrogenase) at collection. Each patient furnished only one value of vitamin D3 (the first dosage).

Data analysis was made using Statistics software, version 5.1/97, considering a 5% level of statistical significance, applying the Fisher, χ^2^ and unpaired* t* tests. Quantitative variables are described by means and standard deviation; absolute (n) and relative (%) frequencies were used for the qualitative variables.

## RESULTS

One hundred patients with cutaneous melanoma were studied, 54 of them men, with a mean age of 54.67 years (22 to 80 years), and 95 Caucasian, between January 2010 and December 2013. Of these 100 patients, 17 presented with the active disease (in lymph nodes and in a distant organ).

The sample can be considered homogeneous, since no differences were observed among the individuals with melanoma with/without activity for the age (p=0.4127-ns), skin color (p=0.1983), and primary site (p=0.4535). However, a statistically superior number was observed in male individuals with non-active melanoma (p=0.0493) (Table 1).

**Table 1:** Biodemographic information

	Melanoma in activity		Melanoma with no activity		Test
Age (years)	n=17		n=83		Unpaired *t* test
Mean ± Standard deviation	57.2±11.0		54.1±14.6		*t*=0.82
Median	60		55		p=0.4127*
Minimum – maximum	41-77		22-83		
Gender	n	%	n	%	χ^2 ^Test
Female	12	70.6	34	41.0	χ^2^_1_=3.86
Male	5	29.4	49	59.0	p=0.0493
Skin color					Fisher’s test
White	15	88.2	80	96.4	p=0.1993*
Non-white	2	11.8	3	3.6	
Primary site					χ^2 ^Test
Limbs	10	58.8	35	42.2	χ^2^_2_=1.58
Trunk	6	35.3	41	49.4	p=0.4535*
Head and neck	1	5.9	7	8.4	

*: no significant.

The mean level of vitamin D3 in the 100 patients with melanoma (25.7ng/mL) was inferior to the recommended levels considered sufficient in clinical practice (>30 ng/L), but was superior to the mean of the control group at HIAE (18.3ng/mL). Of the 100 patients, 69 presented with levels of vitamin D3 <30ng/mL, while in the group of general patients only 8 of the 145 patients presented with sufficient levels of vitamin D3. The vitamin D3 deficiency showed a similar distribution in both groups of patients with active and inactive melanoma: 76.5% and 67.5%, respectively, with p=0.5728 (Table 2).

**Table 2:** Deficiency of 25-hydroxyvitamin D (vitamin D3)

Vitamin D3 deficiency	Melanoma active disease n (%)		Melanoma non-active disease n (%)		Fisher’s test
Yes	13	(76.5)	56	(67.5)	(Fisher’s test)
No	4	(23.5)	27	(32.5)	p=0.5728*

*: no significant.

Applying the unpaired *t* test, the comparison between the individuals of both groups (melanoma with/without activity) demonstrated that the levels of vitamin D3 were statistically superior to those presented by the individuals without melanoma (active: p=0.0134; inactive: p<0.0001).

When comparing the patients with active melanoma and those patients with inactive melanoma, there was no statistical difference (p=0.1824) (Table 3).

**Table 3:** Serum level of 25-hydroxyvitamin D (vitamin D3) – ng/mL

Vitamin D3 (ng/mL)	Melanoma active disease	Melanoma non-active disease	Without melanoma
Patients	n=17	n=83	n=144
Mean ± standard deviation	22.9±9.3	26.5±10.3	18.3±6.8
Median	21	26.8	18
Minimum-maximum	10.79-40.28	5.3-66.4	4-42
Confidence interval 95% of the mean	[18.1-27.6]	[24.2-28.8]	[17.2-19.4]

The Breslow Index proved statistically inferior in the group with inactive melanoma (mean 1.3mm) relative to the group with active melanoma (mean 2.8mm), with p=0.00022 (Table 4).

**Table 4:** Breslow Thickness

Breslow thickness (mm)	Melanoma active disease	Melanoma non-active disease
Patients	n=16*	n=73**
Mean ± standard deviation	2.8±2.7	1.3±1.0
Median	1.8	0.9
Minimum-maximum	0.2-10	0.2-5

* One patient with *in situ* melanoma; **10 patients with *in situ* melanoma.

## DISCUSSION

Melanocytes present with VDR and have the autonomous capacity of synthetizing vitamin D. The expression of VDR in melanoma is more intense than in normal melanocytes.^(10,11)^ Due to the long half-life (more than 250 hours), 25-OH-vitamin D is the best serum indicator of the state of this vitamin in the human body. Calcitriol, the active form of vitamin D, exerts its effects primarily by binding to the nuclear receptor of vitamin D.^(12)^ There still is no definition as to the real role of vitamin D in melanoma.^(13)^ Some sudies reported that polymorphisms of VDR genes are associated to the occurrence of various types of cancer, including melanoma, and that the presence of this polymorphism such as Fokl, Taql, and apa1, could be a biological marker of susceptibility to skin cancer.^(14,15)^

The average level of vitamin D in 100 patients with melanoma (25.7ng/mL) was inferior to the levels recommended as sufficient in clinical practice (>30ng/L), but superior to the mean of the control group at HIAE (18.3ng/mL). Of the 100 individuals studied, 69 presented with levels of vitamin D <30ng/mL. Are the standard levels adequate, and thus, a large portion of the population presents with insufficient levels of vitamin D, including patients with melanoma, or does this standard need to be reevaluated? When we analyze patients with active melanoma (mean=26.5ng/mL) and melanoma with no activity (mean=22.9ng/mL), the vitamin D3 deficiency showed a similar distribution in both groups, with 76.5% and 67.5%, respectively (p=0.1824).

The comparison of the individuals in both groups (melanoma with/without activity) demonstrated that their levels of vitamin D3 are statistically superior to those presented by the individuals without melanoma (active: p=0.0134; inactive: p<0.0001). The bilateral confidence interval of the difference of the means (95%CI) corroborates this information, since it does not include zero. One probable explanation could be that patients with melanoma have habits that lead to greater solar exposure.

Breslow thickness proved statistically inferior in the group with non-active melanoma (median of 0.9mm) when compared to those with active melanoma (median of 1.8mm), with p=0.00022, reinforcing the importance of this measurement as a prognostic factor for cutaneous melanoma.^(7)^

A new light for the role of vitamin D in carcinogenesis emphasizes that once formed in the skin, vitamin D goes into the circulation to perform its physiological functions in the metabolism of calcium and bones. Part of this vitamin D, however, remains in the skin and is activated to interact with the VDR to control the proliferation of cells using a variety of strategies, including interacting with long non-coding RNAs and reducing the risk of photocarcinogenesis.^(16)^

A prospective study evaluating the influence of serum levels of vitamin D on the progression of melanoma found significantly reduced levels in patients with stage IV melanoma when compared to those of stage I (p=0.006). It also identified a tendency towards the presence of a greater Breslow thickness among those with a lower serum level of vitamin D (p=0.078).^(17)^ There is no consensus on clinical recommendation as to the ingestion of vitamin D to prevent skin cancer. Solar exposure for 15 to 20 minutes at least twice a week is sufficient for appropriate synthesis of vitamin D.

## CONCLUSION

In this study, the levels of vitamin D3 in the patients with melanoma were superior to those of the control group formed by patients seen at a general hospital, but inferior to those values recommended as normal in clinical practice. There was no difference in levels of vitamin D3 among the patients with active or no-active melanoma. We did not observe, therefore, a direct relation between high or low levels of vitamin D and the occurrence or gravity of cutaneous melanoma. Studies on the relation between vitamin D and melanoma should be pursued in greater depth.
