# Supplementary material for: Serum level of vitamin D3 in cutaneous melanoma
Source: Einstein (Sao Paulo). 2014 Oct-Dec;12(4):473–6. doi: 10.1590/S1679-45082014AO3090 (PMC4879914; doi:10.1590/S1679-45082014AO3090)
Supplement: Translation

# Translation: Nível sérico de vitamina D3 em portadores de melanoma cutâneo

**Authors:** Renato Santos de Oliveira, Daniel Arcuschin de Oliveira, Vitor Augusto Melão Martinho, Célia Beatriz Gianotti Antoneli, Ludmilla Altino de Lima Marcussi, Carlos Eduardo dos Santos Ferreira

**Affiliations:** 1 Hospital Israelita Albert Einstein, São Paulo, SP, Brasil.; 2 Hospital A.C. Camargo, São Paulo, SP, Brasil.; 3 Universidade Cidade de São Paulo, São Paulo, SP, Brasil.

**Keywords:** Calcifediol, Colecalciferol, Receptores de calcitriol, Deficiência de vitamina D, Melanoma, Sistema imunológico

## Abstract

**Objetivo:**

Comparar o nível de vitamina D3 em portadores de melanoma, em atividade de doença ou não, com os valores de referência e com pacientes de um hospital geral.

**Métodos:**

Os níveis séricos de vitamina D3 foram dosados em portadores de melanoma cutâneo entre 22 a 80 anos, de ambos os sexos, de janeiro de 2010 a dezembro de 2013. As amostras do grupo dos pacientes gerais foram processadas no Hospital Israelita Albert Einstein (grupo controle). A análise dos dados foi realizada utilizando o *software* Statistica.

**Resultados:**

Foram estudados 100 pacientes, sendo 54 homens, com média de idade 54,67 anos, e 95 brancos. Desses 100 pacientes, 17 apresentavam doença em atividade. A média dos níveis de vitamina D3 nos 100 pacientes foi inferior ao nível considerado suficiente, porém acima da média do grupo controle. A deficiência de vitamina D3 apresentou distribuição semelhante nos dois grupos com melanoma (em atividade de doença ou não).

**Conclusão:**

Os níveis de vitamina D3 nos pacientes com melanoma foram superiores aos dos pacientes gerais e inferiores aos de referência. Se os valores de referência estão adequados, grande parte da população apresenta níveis insuficientes de vitamina D3, incluindo os portadores de melanoma, ou tal padrão precisa ser reavaliado. Não houve diferença dos níveis de vitamina D3 entre portadores de melanoma com ou sem atividade. Estudos relacionando vitamina D e melanoma devem ser aprofundados.

## INTRODUÇÃO

O melanoma cutâneo é uma neoplasia maligna, que se origina de melanócitos ou de células precursoras dos mesmos, e que tem alto potencial de metastatização. Embora não seja o câncer de pele mais frequente, é o mais letal. Existem evidências epidemiológicas de que a radiação solar está relacionada à sua carcinogênese. São esperados 5.890 casos novos de melanoma para o ano de 2014 no Brasil.^(1)^

O receptor de vitamina D (VDR, sigla do inglês *vitamin D recepto* *r*) está presente em melanócitos normais e em determinadas linhagens celulares de melanoma.^(2)^ A 25-hidroxivitamina D, também chamada colecalciferol, é normalmente formada na pele a partir de 7-deidrocolesterol, em uma reação fotoquímica catalisada pelo componente ultravioleta da luz solar. A vitamina D3 não é biologicamente ativa, mas convertida por enzimas do fígado e dos rins em 1,25-diidroxicolecalciferol (vitamina D ou calcitriol), um hormônio que regula a absorção de cálcio nos intestinos e os níveis deste nos rins e nos ossos.

Dentre outras funções da vitamina D, há uma extremamente importante, que é a de fortalecer o sistema imunológico. O efeito da vitamina D no sistema imunológico se traduz como um aumento da imunidade inata, associado a uma regulação multifacetada da imunidade adquirida. Já se demonstrou associação entre os níveis deficientes de vitamina D3 e vários tipos de neoplasias malignas, como câncer de colon, de mama e de pele.^(3)^

Vivemos uma controvérsia na comunidade científica sobre quanto de luz solar é apropriado para o equilíbrio entre os efeitos negativos e positivos da exposição à radiação ultravioleta (UV) solar.

A exposição solar é essencial para que a vitamina D seja produzida pela pele, embora a exposição à radiação UV, sem dúvida, seja o fator ambiental mais importante para todos os tipos de câncer dessa localização, por causar danos ao DNA das células da pele. Muitos estudos têm avaliado a importante relação entre a luz solar, a vitamina D e o câncer de pele.^(4,5)^

O melanoma é altamente agressivo, imunogênico e tem habilidade de modular o sistema imunológico em sua própria vantagem. O paciente com melanoma apresenta inúmeros defeitos imunológicos celulares.^(6)^ Dados sugerem que os níveis normais de vitamina D3, no momento do diagnóstico, relacionam-se com melhor prognóstico de pacientes portadores de melanoma cutâneo.^(7,8)^

Apresentamos um estudo transversal que compara os níveis da vitamina D3 em pacientes com melanoma cutâneo, em atividade ou sem, com os níveis normais recomendados pelos laboratórios clínicos e com valores obtidos em pacientes de um hospital geral.

## OBJETIVO

Comparar o nível sérico de 25-hidroxivitamina D (vitamina D3) em pacientes portadores de melanoma cutâneo em atividade de doença e sem atividade com os valores considerados normais para a população geral.

## MÉTODOS

Este trabalho foi devidamente analisado e aprovado pelo Comitê Nacional de Ética em Pesquisa (CONEP), sob o número CAAE: 18762713.9.0000.5492. Todos os pacientes foram orientados quanto ao estudo em questão e assinaram o Termo de Consentimento Livre e Esclarecido.

Os níveis séricos de 25-OH-vitamina D foram dosados em pacientes portadores de melanoma cutâneo, entre 22 e 80 anos, de ambos os sexos, de janeiro de 2010 a dezembro de 2013, tratados e registrados em clínica especializada em oncologia cutânea. Nessa clínica, é realizada, de rotina, a dosagem sérica de 25-OH-vitamina D na admissão de todos os pacientes com diagnóstico de melanoma, bem como em seu seguimento, desde janeiro de 2010.

Os dados dos níveis de vitamina D3 foram coletados a partir dos prontuários identificados com diagnóstico de melanoma, bem como o estado de atividade ou não da doença desses pacientes. As dosagens de vitamina D3 foram realizadas por imunoensaio automatizado, em amostras de soro. Os valores de referência para a vitamina D são determinados por literatura. Atualmente, níveis séricos >30ng/mL (nanogramas por mL) são considerados adequados. Valores entre 10 e 30ng/mL são insuficientes e valores <10ng/mL são considerados deficiência de vitamina D.^(9)^ O grupo controle foi constituido por pacientes gerais cujas amostras para dosagem de vitamina D3 foram processadas pelo laboratório clínico do Hospital Israelita Albert Einstein (HIAE).

A atividade do melanoma (no sítio primário ou em metástases) foi avaliada pelo exame clínico e por exames complementares (tomografia computadorizada de tórax, ultrassonografia de abdome superior e desidrogenase lática) por ocasião da coleta. Cada paciente forneceu apenas um valor de vitamina D3 (sua primeira dosagem).

A análise dos dados foi realizada utilizando o *sof* *tware* Statistica, versão 5.1/97, considerando o nível de significância estatística de 5%, aplicando-se os testes de Fisher, χ^2^ e *t* não pareado. As variáveis quantitativas estão descritas pela média e desvio padrão; frequências absoluta (n) e relativa (%) foram usadas para as variáveis qualitativas.

## RESULTADOS

Foram estudados 100 pacientes portadores de melanoma cutâneno, sendo 54 homens, com média de idade 54,67 anos (22 a 80 anos), e 95 brancos, entre janeiro de 2010 a dezembro de 2013. Destes 100 pacientes, 17 apresentavam doença em atividade (linfonodal e em órgão distante).

A amostra pode ser considerada como homogênea, pois não foram observadas diferenças entre os indivíduos com melanoma em/sem atividade para idade (p=0,4127-ns), cor (p=0,1983) e sítio primário (p=0,4535). Entretanto, foi observado um número estatisticamente superior de indivíduos do sexo masculino com melanoma sem atividade (p=0,0493) (Tabela 1).

**Tabela 1:** Informações biodemográficas

	Melanoma em atividade		Melanoma sem atividade		Teste
Idade (anos)	n=17		n=83		Teste *t* não pareado
Média ± desvio padrão	57,2±11,0		54,1±14,6		*t*=0,82
Mediana	60		55		p=0,4127*
Mínimo - máximo	41-77		22-83		
Gênero	n	%	n	%	Teste χ^2^
Feminino	12	70,6	34	41,0	χ^2^_1_=3,86
Masculino	5	29,4	49	59,0	p=0,0493
Cor					Teste de Fisher
Branco	15	88,2	80	96,4	p=0,1993*
Não branco	2	11,8	3	3,6	
Sítio primário					Teste de χ^2^
Membros	10	58,8	35	42,2	χ^2^_2_=1,58
Tronco	6	35,3	41	49,4	p=0,4535*
Cabeça e pescoço	1	5,9	7	8,4	

*: não significante.

A média dos níveis de vitamina D3 nos 100 pacientes com melanoma (25,7ng/mL) foi inferior aos níveis recomendados como suficientes na prática clínica (>30ng/L), porém esteve acima da média do grupo controle do HIAE (18,3ng/mL). Dos 100 pacientes, 69 apresentaram níveis de vitamina D3 <30ng/mL, enquanto no grupo de pacientes gerais apenas 8 dos 145 pacientes apresentaram níveis suficientes de vitamina D3. A deficiência da vitamina D3 apresentou distribuição semelhante nos dois grupos de pacientes com melanoma em atividade e sem atividade: 76,5% e 67,5%, respectivamente, com p=0,5728 (Tabela 2).

**Tabela 2:** Deficiência da 25-hidroxivitamina D (vitamina D3)

Deficiência vitamina D3	Melanoma em atividade n (%)		Melanoma sem atividade n (%)		Teste de Fisher
Sim	13	(76,5)	56	(67,5)	(Teste de Fisher)
Não	4	(23,5)	27	(32,5)	p=0,5728*

*: não significante.

Aplicando-se o teste *t* não pareado, a comparação dos indivíduos dos dois grupos (melanoma em/sem atividade) demonstrou que os níveis de sua vitamina D3 eram estatisticamente superiores àqueles apresentados pelos indivíduos sem melanoma (em atividade: p=0,0134; sem atividade: p<0,0001).

Quando comparados os pacientes com melanoma em atividade com os pacientes com melanoma sem atividade, não houve diferença estatística significante (p=0,1824) (Tabela 3).

**Tabela 3:** Nível sérico de 25-hidroxivitamina D (vitamina D3) – ng/mL

Vitamina D3 (ng/mL)	Melanoma em atividade	Melanoma sem atividade	Sem melanoma
Pacientes	n=17	n=83	n=144
Média ± desvio padrão	22,9±9,3	26,5±10,3	18,3±6,8
Mediana	21	26,8	18
Mínimo-máximo	10,79-40,28	5,3-66,4	4-42
Intervalo de confiança 95% da média	[18,1-27,6]	[24,2-28,8]	[17,2-19,4]

O índice de Breslow se mostrou estatisticamente inferior no grupo com melanoma sem atividade (média de 1,3mm) em relação ao grupo portador de melanoma em atividade (média de 2,8mm), com p=0,00022 (Tabela 4).

**Tabela 4:** Espessura de Breslow

Espessura de Breslow (mm)	Melanoma em atividade	Melanoma sem atividade
Pacientes	n=16*	n=73**
Média ± desvio padrão	2,8±2,7	1,3±1,0
Mediana	1,8	0,9
Mínimo-máximo	0,2-10	0,2-5

* Um pacientes com melanoma *in situ*; **10 pacientes com melanoma *in situ*.

## DISCUSSÃO

O melanócitos apresentam VDR e têm capacidade autônoma de sintetizar a vitamina D. A expressão dos VDR, no melanoma, é mais intensa do que nos melanócitos normais.^(10,11)^ Devido à longa meia-vida (mais de 250 horas) a 25-OH-vitamina D é o melhor indicador sérico do estado dessa vitamina no organismo humano. O calcitriol, a forma ativa da vitamina D, exerce seus efeitos principalmente ligando-se ao receptor nuclear de vitamina D.^(12)^ Ainda não existe uma definição sobre o real papel da vitamina D no melanoma.^(13)^ Estudos reportam que polimorfismo de genes do VDR estão associados à ocorrência de diversos tipos de câncer, incluindo o melanoma, e que a presença desse polimorfismo como Fokl, Taql e apa1 poderia ser um marcador biológico de suscetibilidade ao câncer de pele.^(14,15)^

A média dos níveis de vitamina D nos 100 pacientes com melanoma (25,7ng/mL) foi inferior aos níveis recomendados como suficientes na prática clínica (>30ng/L), porém acima da média do grupo controle do HIAE (18,3ng/mL). Dos 100 estudados, 69 apresentaram níveis de vitamina D <30ng/mL. Será que os valores padrões estão adequados e, assim, grande parte da população apresenta níveis insuficientes de vitamina D, incluindo os pacientes portadores de melanoma, ou tal padrão precisa ser reavaliado? Quando analisamos os pacientes portadores de melanoma em atividade (média=26,5ng/mL) e sem atividade (média=22,9ng/mL), a deficiência da vitamina D3 apresentou distribuição semelhante nos dois grupos, com 76,5% e 67,5%, respectivamente (p=0,1824).

A comparação dos indivíduos dos dois grupos (melanoma em/sem atividade) demonstrou que os níveis de sua vitamina D3 são estatisticamente superiores àqueles apresentados pelos indivíduos sem melanoma (em atividade: p=0,0134; sem atividade: p<0,0001). O intervalo de confiança bilateral da diferença das médias (IC95%) corrobora essa informação, pois os mesmos não incluem o zero. Uma provável explicação seria que os pacientes com melanoma têm hábitos que levam à maior exposição solar.

A espessura de Breslow se mostrou estatisticamente inferior no grupo com melanoma sem atividade (mediana de 0,9mm) comparada com aqueles com melanoma em atividade (mediana de 1,8mm), com p=0,00022, reforçando a importância dessa medida como fator prognóstico do melanoma cutâneo.^(7)^

Uma nova luz para o papel da vitamina D na carcinogênese ressalta que, uma vez formada na pele, a vitamina D vai para a circulação para realizar suas funções fisiológicas no metabolismo do cálcio e dos ossos. Parte dessa vitamina D, no entanto, permanece na pele e é ativada para interagir com seu VDR para controlar a proliferação de células, utilizando uma variedade de estratégias, incluindo a de interagir com longos RNAs não codificantes, e reduzindo o risco de fotocarcinogénese.^(16)^

Estudo prospectivo avaliando a influência dos níveis séricos de vitamina D na evolução do melanoma encontrou níveis significativamente reduzidos nos pacientes com melanoma estádio IV quando comparados com os de estádio I (p=0,006). Encontrou também uma tendência da presença de espessura de Breslow maior entre aqueles com menor nível sérico de vitamina D (p=0,078).^(17)^ Não há consenso sobre recomendação clínica de ingestão de vitamina D para prevenir câncer de pele. Uma exposição solar de 15 a 20 minutos pelo menos duas vezes por semana é suficiente para a síntese apropriada de vitamina D.

## CONCLUSÃO

Neste estudo, os níveis de vitamina D3 nos pacientes com melanoma foram superiores àqueles do grupo controle formado por pacientes atendidos em hospital geral, mas inferiores aos valores padronizados como normais na prática clínica. Não houve diferença dos níveis de vitamina D3 entre os portadores de melanoma com ou sem atividade da doença. Não observamos, portanto, uma relação direta entre níveis altos ou baixos de vitamina D e a ocorrência ou gravidade do melanoma cutâneo. Estudos sobre a relação entre vitamina D e melanoma devem ser aprofundados.
